# Plasma anti‐BIRC5 IgG may be a useful marker for evaluating the prognosis of nonsmall cell lung cancer

**DOI:** 10.1002/2211-5463.12417

**Published:** 2018-04-16

**Authors:** Huan Zhao, Xuan Zhang, Zhifeng Han, Zhenqi Wang, Yao Wang

**Affiliations:** ^1^ Jilin Provincial Key Laboratory on Molecular and Chemical Genetics Second Hospital of Jilin University Changchun China; ^2^ China‐Japan Union Hospital Jilin University Changchun China; ^3^ School of Public Health Jilin University Changchun China

**Keywords:** autoantibodies, BIRC5, MYC, NSCLC, tumor immunity

## Abstract

A recent study demonstrated that circulating levels of IgG antibodies against linear peptide antigens derived from baculoviral IAP repeat‐containing protein 5 isoform 2 (BIRC5) and myc proto‐oncogene protein (MYC) were significantly increased in nonsmall cell lung cancer (NSCLC). This study was undertaken to replicate this initial work in an independent sample. An enzyme‐linked immunosorbent assay (ELISA) was developed in‐house to examine plasma IgG antibodies for three linear peptide antigens derived from BIRC5a, BIRC5b, and MYC in 211 patients with NSCLC and 200 control subjects. A Mann–Whitney *U*‐test demonstrated that plasma anti‐BIRC5a IgG levels, but not anti‐BIRC5b or anti‐MYC IgG levels, were significantly higher in NSCLC patients than control subjects, especially in male patients. Both squamous cell cancer and adenocarcinoma showed increased anti‐BIRC5a IgG levels, but the IgG levels were not found to be changed significantly in the early stage of NSCLC. Kaplan–Meier survival analysis showed that NSCLC patients with high anti‐BIRC5b IgG levels had better prognosis and longer overall survival (OS) than patients with low anti‐BIRC5b IgG levels, although this significant difference failed to survive the adjustment for age, gender, NSCLC stages, and types. Plasma anti‐BIRC5a and MYC IgG levels did not show significant associations with OS. In conclusion, Plasma anti‐BIRC5 IgG may be a useful marker for assessment of prognosis of NSCLC but not for early diagnosis of this malignancy.

**Abbreviations**
BIRC5: baculoviral IAP repeat containing 5
CT: computed tomography
CV: coefficient of variation
ELISA: enzyme‐linked immunosorbent assay
IgG: immunoglobulin G
MYC: myc proto‐oncogene protein
NC: negative control
NSCLC: nonsmall cell lung cancer
OD: optical density
OS: overall survival
PBS: phosphate‐buffered saline
PC: positive control
QC: quality control
SBR: specific binding ratio
SD: standard deviation
TAA: tumor‐associated antigen
TNM: tumor, node, and metastasis

Lung cancer is one of the most fatal malignant tumors 1 and a leading cause of cancer‐related death 2. There are nearly 11 million new cases diagnosed as having lung cancer worldwide each year 3. A recent report by the National Cancer Center, China, indicated that lung cancer was also the first cause of cancer death in China; in 2013, about 733 000 cases were newly diagnosed with lung cancer and about 591 000 cases that died of lung cancer 4. Nonsmall cell lung cancer (NSCLC) is the most common type of lung cancer, accounting for 80–85% of all cases with this malignancy 5.

The high mortality rate of lung cancer is largely attributable to late diagnosis 6. If this malignant tumor was identified at stage 1, the 5‐year survival rate can rise from less than 15% on average to 50% 7. Currently, computed tomography (CT) has widely been used for screening of lung cancer among the population, but its shortcoming such as low specificity 8, radiation, and high cost has limited its clinical application 9. So there is an urgent need to develop a powerful technology for diagnosis of lung cancer at an early stage.

Tumor development is often accompanied with the overexpression of tumor‐associated antigens (TAAs) and altered protein structure due to mutation. These alterations may elicit immune responses resulting in the production of anti‐TAA autoantibodies. Moreover, some publications reported that anti‐TAA autoantibodies could be detected before symptomatic cancer was developed 10, 11. Thus, detection of circulating anti‐TAA autoantibodies is a promising method for early screening of malignant diseases.

A recent study demonstrated that plasma levels of IgG antibodies against linear peptide antigens derived from baculoviral IAP repeat‐containing protein 5 isoform 2 (BIRC5) and myc proto‐oncogene protein (MYC) were found to be significantly increased in NSCLC 12. Because the synthesis of linear peptides is more economical than the production of recombinant TAAs, the test developed with linear peptide antigens would be more suitable for large‐scale screening of individuals at high risk of lung cancer. For this reason, this study was designed to replicate the initial finding independently in a large sample size and to explore the relationship between circulating antibody levels and survival time in patients with NSCLC.

## Materials and methods

### Subjects

A total of 411 archived plasma samples collected from patients with NSCLC and control subjects were used to detect circulating levels of anti‐BIRC5 and anti‐MYC IgG antibodies. Their demographic information is given in Table 1. These patients were admitted to the Department of Thoracic Surgery, China‐Japan Union Hospital of Jilin University, in the period between November 2012 and August 2016. Their diagnosis and tumor stages were made based on radiographic examination and histological confirmation. Eligible patients for this study were restricted to those with adenocarcinoma and squamous cell carcinoma only. Based on the TNM (tumor, node, and metastasis) staging information, these patients were classified into four groups: group I for stage T_1_N_0_M_0_,group II for stage T_1_N_1_M_0_ + T_2_N_0_M_0_,group III for stage T_2_N_1_M_0_ + T_3_N_0_M_0_, and group IV for stages 3 and 4. Their blood samples were taken prior to any anticancer treatment. Clinical information and follow‐up data were obtained from the Large‐scale Data Analysis Center of Cancer Precision Medicine‐LinkDoc database and analyzed using data technology support from LinkDoc 13. The control subjects were recruited from local communities at the same time as sample collection from patients with NSCLC and well matched with patient subjects on age, gender, and smoking history (Table 1). These control subjects underwent clinical interview and radiographic or imaging examination to exclude those subjects who had any type of malignant tumors and a severe form of autoimmune diseases such as autoimmune thyroid disease, pernicious anemia, type 1 diabetes, celiac disease, multiple sclerosis, systemic lupus erythematosus, and inflammatory bowel diseases. All subjects were of Chinese Han origin, and all gave written informed consent to donate blood samples for study of the pathogenesis of lung cancer. This work was approved by the Ethics Committee of Second Hospital of Jilin University and conformed to the Declaration of Helsinki.

**Table 1:** Demographic information of NSCLC patients and control subjects

Characteristic	Patients	Controls
Age (years)	58.7 ± 8.7	58.6 ± 9.3
Gender		
Male	131 (62.1%)	103 (48.5%)
Female	80 (37.9%)	97 (51.5%)
Smoking history	106 (50.2%)	91 (45.5%)
Type of tumor		
Squamous cell carcinoma	87 (41.2%)	N/A
Adenocarcinoma	124 (58.8%)	N/A

### Detection of plasma IgG levels

Three linear peptide antigens were used to develop an in‐house enzyme‐linked immunosorbent assay (ELISA) for detection of circulating IgG antibodies against BIRC5 and MYC. Of the three peptide antigens that were synthesized by solid‐phase chemistry with a purity of >95%, BIRC5a and MYC were described in previous publications 14 and BIRC5b was designed using a computational epitope prediction software (http://www.iedb.org). Their sequences are given in Table 2. The in‐house ELISA was developed based on a recent publication 15, 16. In brief, each synthetic peptide antigen was dissolved in 67% acetic acid to obtain a concentration of 5 mg·mL^−1^ as stock solution and diluted just before use in coating buffer that was 0.1 m phosphate buffer containing 0.15 m NaCl and 10 mm EDTA, pH 7.2. Maleimide‐activated plates (Cat. 15150, Thermo Scientific, Rockford, IL, USA) were coated with synthetic peptide antigens based on the manufacturer's instruction and stored at −4 °C until use within 6 months.

**Table 2:** Information of peptide antigens derived from BIRC5 and MYC

Antigens	Sequence(N→C)	NCBI accession	Position (aa)
BIRC5a	H‐dflkdhristfknwlhhfqglfpgatslpv‐OH	NP_001012270	13–24 + 115–131
BIRC5b	H‐epdddpmqrkptirrknlrklrrkcavpss‐OH	NP_001012270	68–97
MYC	H‐rvkldsvrvlrqisnnrkcfellptpplsps‐OH	NP_002458	68–79 + 339–357

Plasma sample and positive control (PC) sample diluted 1 : 100 in assay buffer that was phosphate‐buffered saline (PBS) containing 0.5% bovine serum albumin was added to each sample well, and 50 μL assay buffer to each negative control (NC) well. Following incubation for 1.5 h at room temperature, the plate was washed three times with 200 μL wash buffer that PBS containing 0.1% Tween 20, and 50 μL peroxidase‐conjugated goat anti‐human IgG antibody (ab98567, Abcam, Cambridge, UK) diluted 1 : 50 000 in assay buffer was added to each well. After incubation at room temperature for 1 h, color development was initiated by adding 50 μL stabilized chromogen (SB02, Life Technologies, Frederick, MD, USA) and terminated after 30 min by adding 25 μL stop solution (SS04, Life Technologies, Frederick, MD, USA). The measurement of optical density (OD) was completed on a microplate reader within 15 min at 450 nm with a reference wavelength of 620 nm. All the samples were tested in duplicate, and the specific binding ratio (SBR) was used to represent the relative levels of plasma IgG antibodies. Calculation of SBR is as follows: SBR = (OD_sample_−OD_NC_)/(OD_PC_−OD_NC_).

The inter‐assay deviation was estimated using quality control sample, which was randomly collected from >100 unrelated healthy subjects, and the coefficient of variation (CV) was used to represent reproducibility of the in‐house ELISA.

Total IgG levels in plasma were detected with Human Uncoated ELISA Kit with Plates (Cat. 88‐50550, Thermo Scientific) based on the manufacturer's instruction.

### Data analysis

Kolmogorov–Smirnov test was used to examine a normal distribution of circulating IgG levels tested in both the patient group and the control group, respectively. Because plasma anti‐BIRC5a, anti‐BIRC5b, and anti‐MYC IgG levels showed a skewed distribution (Table 3), Mann–Whitney *U*‐test was thus applied to examine the difference in their plasma IgG levels and Student's *t‐*test was used to examine the difference in total IgG levels between the two groups. Medians were used as a cutoff to determine a low IgG level that was defined as the SBR below a median and a high IgG level that was defined as the SBR above the median. Kaplan–Meier survival analysis was applied to assess the overall survival (OS) that was defined as the period between the date of first hospitalization and that of death or censoring. Cox regression was applied to examine the difference in OS between patients with low IgG levels and those with high IgG levels.

**Table 3:** Kolmogorov–Smirnov test for a normal distribution of plasma IgG levels

Antibody	Skewness	Kurtosis	*P*
BIRC5a			
Patient	0.943	0.584	0.047
Control	1.206	2.008	0.001
BIRC5b			
Patient	2.451	8.817	<0.001
Control	1.951	6.85	0.02
MYC			
Patient	1.295	2.504	0.058
Control	1.615	4.027	0.002
Total IgG			
Patient	0.193	−0.326	0.952
Control	0.225	−0.414	0.327

## Results

The in‐house ELISA showed a good reproducibility with a CV of 12.7% from anti‐BIRC5a IgG assay, 10.6% from anti‐BIRC5b IgG assay, and 11.9% from anti‐MYC IgG assay.

Mann–Whitney *U*‐test demonstrated that plasma anti‐BIRC5a IgG levels were significantly higher in patients with NSCLC than control subjects (*Z* = −4.06, *P < *0.001), and male patients mainly contributed to the increased anti‐BIRC5 IgG levels in NSCLC (*Z* = −4.16, *P < *0.001); however, neither anti‐BIRC5b IgG nor anti‐MYC IgG levels showed a significant change in patients with NSCLC compared with controls subjects (Table 4). As shown in Table 5, both squamous cell cancer and adenocarcinoma contributed to increased anti‐BIRC5a IgG levels in NSCLC patients. As shown in Table 6, anti‐BIRC5a IgG level was significantly increased in groups II, III, and IV but not in group I, when compared with control subjects. There was no significant difference in total IgG level between the patient group (3.00 ± 1.14 mg·mL^−1^) and the control group (3.10 ± 1.08 mg·mL^−1^, *t *=* *0.85, *P *=* *0.396).

**Table 4:** The levels of plasma IgG against BIRC5 and MYC in patients with NSCLC and control subjects

IgG	Group	Patient (*n*)	Control (*n*)	*Z* a	*P* b
BIRC5a	Male	1.35 ± 0.52 (131)	1.10 ± 0.48 (103)	−4.16	<0.001
	Female	1.28 ± 0.55 (80)	1.18 ± 0.54 (97)	−1.31	0.19
	Both	1.33 ± 0.53 (211)	1.14 ± 0.51 (200)	−4.06	<0.001
BIRC5b	Male	1.04 ± 0.52 (131)	0.96 ± 0.48 (103)	−1.34	0.18
	Female	1.01 ± 0.64 (80)	1.02 ± 0.66 (97)	−0.18	0.86
	Both	1.03 ± 0.57 (211)	0.99 ± 0.58 (200)	−1.19	0.23
MYC	Male	1.07 ± 0.52 (131)	1.07 ± 0.53 (103)	−0.09	0.93
	Female	0.99 ± 0.47 (80)	1.09 ± 0.58 (97)	−0.98	0.33
	Both	1.04 ± 0.50 (211)	1.08 ± 0.55 (200)	−0.66	0.51

Plasma IgG levels are expressed as mean ± SD in SBR.

a Mann–Whitney *U*‐test (two‐tailed).

b *P *<* *0.07 was considered to be statistically significant as three individual antigens were tested.

**Table 5:** The level of plasma IgG antibodies against BIRC5 and MYC in two histological types of NSCLC

IgG	Patient (*n*)	Control (*n*)	*Z* a	*P* b
BIRC5a				
Squamous	1.41 ± 0.54 (87)	1.14 ± 0.51 (200)	−4.46	<0.001
Adenocarcinoma	1.26 ± 0.52 (124)	1.14 ± 0.51 (200)	−2.45	0.014
BIRC5b				
Squamous	1.03 ± 0.57 (87)	0.99 ± 0.58 (200)	−1.10	0.271
Adenocarcinoma	1.03 ± 0.57 (124)	0.99 ± 0.58 (200)	−0.88	0.377
MYC				
Squamous	1.08 ± 0.47 (87)	1.08 ± 0.55 (200)	−0.46	0.65
Adenocarcinoma	1.01 ± 0.52 (124)	1.08 ± 0.55 (200)	−1.33	0.18

Plasma IgG levels are expressed as mean ± SD in SBR.

Mann–Whitney *U*‐test (two‐tailed).

*P *<* *0.07 was considered to be statistically significant as three individual antigens were tested.

**Table 6:** The level of circulating antibodies against BIRC5 and MYC in four groups of NSCLC

TAAs	Group^a^	Patient (*n*)	Control (*n*)	*Z* b	*P* c
BIRC5a	I	1.27 ± 0.76 (20)	1.14 ± 0.51 (200)	−0.23	0.82
	II	1.32 ± 0.49 (101)	1.14 ± 0.51 (200)	−3.60	<0.001
	III	1.43 ± 0.57 (41)	1.14 ± 0.51 (200)	−3.26	0.001
	IV	1.26 ± 0.48 (49)	1.14 ± 0.51 (200)	−2.07	0.04
BIRC5b	I	0.98 ± 0.45 (20)	0.99 ± 0.58 (200)	−0.29	0.77
	II	1.10 ± 0.65 (101)	0.99 ± 0.58 (200)	−1.68	0.09
	III	0.99 ± 0.45 (41)	0.99 ± 0.58 (200)	−0.71	0.48
	IV	0.94 ± 0.51 (49)	0.99 ± 0.58 (200)	−0.29	0.77
MYC	I	0.94 ± 0.61 (20)	1.08 ± 0.55 (200)	−1.49	0.14
	II	1.03 ± 0.54 (101)	1.08 ± 0.55 (200)	−1.08	0.28
	III	1.11 ± 0.45 (41)	1.08 ± 0.55 (200)	−0.92	0.36
	IV	1.04 ± 0.43 (49)	1.08 ± 0.55 (200)	−0.02	0.98

Plasma IgG levels are expressed as mean ± SD in SBR.

a Group I for stage T_1_N_0_M_0_, group II for stage T_1_N_1_M_0_+T_2_N_0_M_0_, group III for stage T_2_N_1_M_0_+T_3_N_0_M_0_, and group IV for stages 3 and 4.

b Mann–Whitney *U*‐test (two‐tailed).

c *P *<* *0.017 was considered to be statistically significant as three individual antigens were tested.

**Figure 1:**
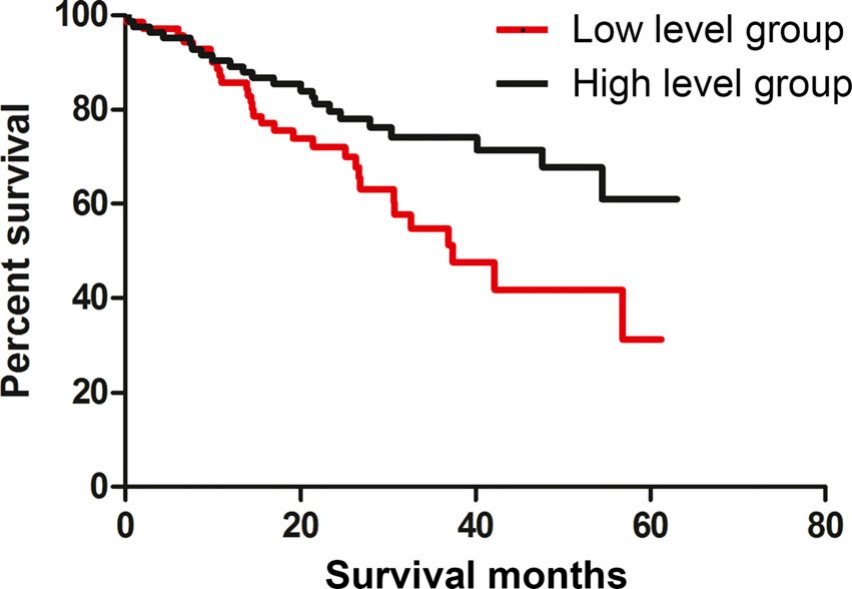
Kaplan–Meier survival analysis for anti‐BIRC5b IgG levels and overall survival (OS) in patients with NSCLC.

Until the end of 2017, 154 patients were successfully followed up, 52 of whom had died. As shown in Fig. 1, Kaplan–Meier survival analysis showed a mean OS of 50.8 (±2.41 standard error, SE) months in NSCLC patients with a high anti‐BIRC5b IgG level as compared to 39.6 (±2.92 SE) months in those patients with a low anti‐BIRC5b IgG level (χ^2^ = 6.7, *P *=* *0.01), although this significant difference failed to survive the correction of a false‐positive rate (type I errors) for age, gender, stages, and types of NSCLC (corrected *P *>* *0.05). Plasma anti‐BIRC5a and MYC IgG levels did not show a significant association with OS (Table 7).

**Table 7:** Kaplan–Meier survival analysis of differences in overall survival (OS) between patients with low IgG levels and those with high IgG levels

IgG	OS (months)^a^			
	Low‐level group	High‐level group	χ^2^ b	*P* c
BIRC5a	47.6 ± 2.66	44.3 ± 2.82	1.0	0.32
BIRC5b	39.6 ± 2.92	50.8 ± 2.41	6.7	0.01
MYC	46.1 ± 2.61	45.9 ± 2.87	0.03	0.86

a Mean ± SE in OS.

b Calculated from Cox regression analysis.

c Uncorrected *P*‐value for age, gender, NSCLC stages, and types.

## Discussion

Baculoviral IAP repeat containing 5, also called survivin, is a unique member of the inhibitor‐of‐apoptosis gene family 17. It is often present in fetal development, transformed cells, and tumors, but absent in most normally differentiated adult tissues 18. Unlike the family of Bcl‐2 molecules, survivin inhibits apoptosis through both the death‐receptor pathway and the mitochondrial pathway 19. In addition, survivin also plays a critical role in regulating cell division. Therefore, survivin is likely to be involved in tumorigenesis.

Circulating antibodies to survivin have been found to be elevated in a range of malignant tumors, such as ovarian cancer 20, cervical cancer 14, gastric cancer 21, colorectal cancer 22, and lung cancer 23. In this study, we found that plasma anti‐BIRC5a IgG levels were significantly higher in patients with NSCLC than control subjects (Table 4). While our results of altered anti‐BIRC5 IgG levels in NSCLC are consistent with the previous studies 12, 24, this IgG antibody cannot serve as a biomarker for early diagnosis of NSCLC as plasma anti‐BIRC5 IgG levels were not significantly changed in patients with early‐stage NSCLC (Table 6). It is worth noting that circulating anti‐BIRC5 IgG may have a prognostic value as NSCLC patients with high anti‐BIRC5b IgG levels survived longer than those with low anti‐BIRC5b IgG levels (Table 7 and Fig. 1). Possibly, anti‐BIRC5b IgG has a protective role in suppressing the development of NSCLC progression. Moreover, plasma anti‐MYC IgG level was reported to be increased in patients with NSCLC in a previous study 12, but we failed to replicate this initial finding in the present work. This inconsistency may be due to either application of different ELISA methods, small power, or skewed sampling. Our sample size used in this study is much larger than the previous report that recruited only 109 patients with NSCLC 12, so that our finding may be more reliable.

This is our first attempt to identify a specific peptide epitope derived from the human survivin. The present study demonstrated that BIRC5a is more immunogenic than BIRC5b although anti‐BIRC5b IgG is more important for survival of NSCLC patients, suggesting that mapping of powerful epitopes is particularly important to develop a more sensitive antibody test. In fact, each protein may carry many epitopes that may be recognized by T‐independent B1 cells and produce autoantibodies 25. Different epitopes have different binding affinities to B‐cell receptors (BCRs) and induce different immune responses. So the antibody test developed with linear peptide epitopes may be more accurate for detection of specific antibodies than that developed with recombinant proteins. Further investigation will be carried out to compare the difference in specificity and accuracy between antibody tests with two different antigen systems.

## Research involving human participants and/or animals

This work was approved by the Ethics Committees of the ethics committee of the Second Hospital of Jilin University, Changchun, China, and performed in accordance with the ethical standards as laid down in the 1964 Declaration of Helsinki and its later amendments.

## Informed consent

All subjects recruited for this study gave written informed consent to participate in this study.

## Author contributions

HZ and ZW mainly performed laboratory work, data analysis, and preparation of the manuscript. XZ conceived of this study and corrected the manuscript. ZH and YW were mainly responsible for identification of patients, correction of blood samples, and clinical information.
